# Local human impacts interact with geography to drive benthic community depth zonation on contemporary coral reefs

**DOI:** 10.1098/rspb.2024.1885

**Published:** 2025-06-11

**Authors:** Rebecca J. Turner, Laura E. Richardson, Courtney S. Couch, Jessica A. Harvey, Gareth J. Williams

**Affiliations:** ^1^ School of Ocean Sciences, Bangor University, Bangor, Anglesey, UK; ^2^ ARC Centre of Excellence for Coral Reef Studies, Townsville, Queensland, Australia; ^3^ Cooperative Institute for Marine and Atmospheric Research, School of Ocean and Earth Science and Technology, Honolulu, HI, USA; ^4^ Pacific Islands Fisheries Science Center, National Marine Fisheries Service, National Oceanic and Atmospheric Administration, Honolulu, HI, USA

**Keywords:** anthropogenic impacts, benthic assemblages, depth strata, Pacific Ocean, spatial heterogeneity, vertical zonation

## Abstract

Changes in biophysical conditions and energetic resource supply across depths are predicted to promote or limit the abundance of different coral reef benthic groups. However, the degree to which regional differences in biophysical processes govern and local human activities might alter naturally occurring depth zonation patterns remains unclear. Here, we used 2239 reef surveys conducted between 0 and 30 m depth around 33 islands (18 unpopulated and 15 populated) across the Pacific Ocean to quantify the percentage cover change of seven broad benthic groups. We tested whether natural depth zonation patterns differed across geographies (using six ecoregions) and whether and how local human impacts might disrupt these natural zonation patterns. We found benthic community depth zonation did not always occur. At the three ecoregions where depth zonation existed, there was no universal ‘natural’ zonation pattern and the benthic groups most responsible for driving patterns of depth zonation differed across geographies. We also found evidence of human-disrupted changes to benthic community depth zonation; patterns were inversed across depths and less distinct at populated compared to unpopulated islands within two ecoregions. We show coral reef communities are naturally highly variable and that human activities can disrupt natural patterns of ecological organization in contemporary ecosystems.

## 1. Introduction

The structure of ecological communities is strongly coupled with their surrounding biophysical environment [1,2]. The ‘niche optimum’ theory predicts that species best adapted to the surrounding environment will become the most dominant [3,4], with ecological organization determined by inter- and intraspecific competition for space and food [5,6]. Foundational models of ecological theory captured community patterns of zonation and their key biophysical drivers across numerous natural systems, including zonation in vegetation communities with increasing altitude on mountains [7] and vertical distribution changes in epiphytic lichen communities in trees [8]. These models were then tested and adapted to capture vertical and horizontal zonation patterns in marine systems, including inter-tidal temperate rocky shore communities [9,10] and subtidal kelp forest communities [11].

On tropical coral reefs, depth has long been acknowledged as a key determinant of benthic community zonation [12–15]. Along the depth gradient, there are predictable changes in biophysical conditions [16–18] and energetic resource supply [19–21] that either promote or limit the abundance of different reef community members. In the well-lit but wave-exposed shallows, there is abundant photosynthetic active radiation supporting autotrophic and more wave-tolerant benthic organisms, such as low-lying fleshy turf algae and crustose coralline algae (CCA) [22–24]. In response to this, there is often a higher biomass of primary consumer fishes (herbivores) that capitalize on these increased algal food sources in the shallows relative to deeper depths [25]. With increasing depth, the loss of light is replaced by an increased proximity to upwelled sources of nutrients and enhanced planktonic production supporting heterotrophic organisms such as planktivorous fishes [25,26] and larger macroalgae that capitalize on the reprieve from surface wave stress and increased nutrient concentrations [24,27]. Mixotrophic organisms with flexible trophic strategies, such as many symbiotic reef-building corals, capitalize on both the autotrophic and heterotrophic food sources and occupy space throughout the depth gradient [28]. They do, however, have predictable changes in morphologies with depth, with more wave-tolerant encrusting corals occupying the shallows, and more structurally complex corals that are vulnerable to dislodgement by waves occupying deeper depths [17,24,29,30]. Despite these advances in ecological theory, many zonation paradigms were derived from geographically limited studies and occurred prior to the rapid escalation of cross-scale human impacts that have restructured coral reefs in recent decades.

Globally, ecosystems are being rapidly disordered by the impacts of anthropogenic stressors, with tropical marine communities exhibiting some of the steepest rates of change [31,32]. In addition to the global impacts of human-induced climate change, coral reefs face myriad local human impacts such as overfishing, coastal development, sedimentation and nutrient enrichment [33–35]. These disturbances can alter the outcomes of benthic organism competitive interactions [36], often in favour of non-accreting benthic organisms like fleshy algae at the demise of reef-building corals [37,38]. In doing so, human impacts can reconfigure natural patterns of reef community organization across scales [39–41]. Although the effects of natural environmental gradients are still present, evidence is now emerging that local human impacts can overwhelm their influence and instead become the dominant driving force of reef community organization [40,41]. As such, the degree to which historic ecological paradigms can capture patterns of ecological organization in contemporary coral reef ecosystems has come into question [25,42].

Across the Pacific Ocean, reef community structure also varies between ecoregions even in the absence of local human impacts [38,43,44]. Across these geographies, there are gradients in surface wave energy, seawater temperature, irradiance and primary production [45,46], and reefs have adapted to thrive in their region-specific, climatological range. For example, in the absence of local human impacts, the cover of reef-building corals and CCA increases with increasing primary production [40]. In the higher latitude unpopulated northwestern Hawaiian Islands, some reefs support naturally high levels of macroalgal cover and low reef-building coral cover [47] owing to lower aragonite saturation states that are less ideal for coral growth [48]. These gradients in biophysical drivers across ecoregions therefore set natural bounds on reef community organization and the basis for what changes may, or may not, occur under local human-induced change. It is therefore reasonable to expect that patterns of benthic community depth zonation may vary in the absence or presence of local human impacts across different geographies. However, we currently have a very limited understanding of whether and how these interacting forces govern depth zonation patterns of reef organisms [25].

Here, we used 2239 individual reef surveys conducted between 2010 and 2014 around 33 islands and atolls (18 unpopulated and 15 populated) across the Pacific Ocean to: (i) quantify the natural depth zonation patterns of coral reef benthic communities in the absence of confounding local human impacts across three depth strata (shallow: 0−6 m, mid: >6−18 m, deep: >18−30 m); (ii) test whether these natural depth zonation patterns differ across geographies (using six ecoregions spanning 50 degrees of latitude and 40 degrees of longitude); and (iii) test whether and how local human impacts disrupt these natural depth zonation patterns on contemporary reefs.

## 2. Methods

### (a) Sampling design

**Figure 1.**
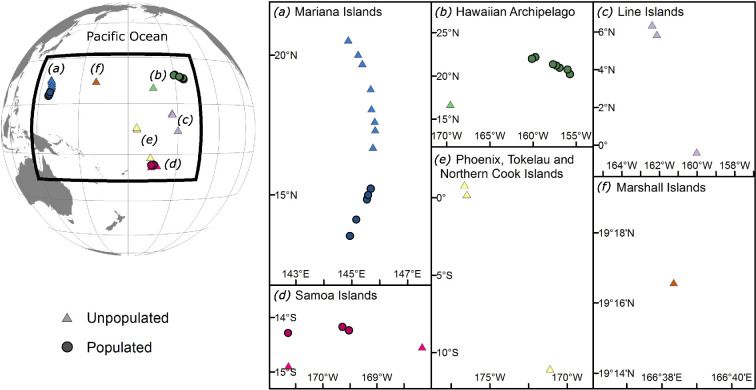
Our 33 study islands within six marine ecoregions across the Pacific Ocean. Benthic community data were collected using 2239 reef surveys at unpopulated (triangles; *n* = 18) and populated (circles; *n* = 15) islands.

Benthic surveys were conducted at 33 islands across the United States-affiliated Pacific as part of the National Oceanic and Atmospheric Administration (NOAA) Coral Reef Conservation Program’s National Coral Reef Monitoring Program (NCRMP) (figure 1). These islands span broad geographies and large environmental and anthropogenic gradients known to influence benthic community structure on coral reefs [40,45]. In our analysis, we used the Marine Ecoregions of the World classification system [49], where ecoregions were defined as areas of relatively homogeneous species composition, probably determined by oceanographic or topographic features and distinct biogeographic forcing agents. As this was an ecological study and we were testing whether natural depth zonation patterns differ across geographies, we found this classification system more appropriate than geopolitical boundaries (see the electronic supplementary material ST1 for details on ecoregion/geopolitical region substitutions).

Surveys were conducted across six marine ecoregions across the Pacific: Hawaii Islands, Mariana Islands, Marshall Island, Phoenix, Tokelau and Northern Cook (PTNC) Islands, Line Islands and Samoa Islands (figure 1). These ecoregions include islands that are ‘unpopulated’ and ‘populated’ by humans, except the Line Islands, Marshall Island and PTNC Islands which are only unpopulated. We defined population status (populated versus unpopulated) using a combination of local human population density and the island’s proximity to larger population centres. ‘Unpopulated’ islands have low to negligible direct local human impacts and are located more than 100 km from the nearest human settlement comprising greater than 50 people. ‘Populated’ islands are populated by more than 50 people and exposed to direct local impacts from human activities (see the electronic supplementary material S1.1 for details on island population status classification). Unpopulated (*n* = 18) and populated islands (*n* = 15) were used here to compare ‘natural’ benthic distribution patterns in the absence of direct local human impacts versus those exposed to ongoing direct anthropogenic disturbances, such as fishing and coastal development (*sensu* Williams *et al.* [40]). As local human population density is often a poor predictor of gradients in local human impacts on coral reefs [50], including at some of our study islands (e.g. Hawaii, [35]), we used this binary split as this ensured we were either comparing *some* or *no* local human impacts, instead of trying to quantify island-to-island gradients in impact. See the electronic supplementary material ST2 for the number of unpopulated and populated islands within each ecoregion. The unpopulated northwestern Hawaiian Islands commonly surveyed by NCRMP were not included in this study as the benthic community structure had not been quantified at the time of analysis.

Within each ecoregion, survey site locations around each island were pre-selected using the NCRMP stratified random sampling design [51]. Using geographical information system substrate and strata maps, sites were randomly selected within hard bottom forereef habitats (reef slope facing the open ocean) stratified by three depth strata (shallow: 0−6 m, mid: >6–18 m, and deep: >18–30 m). The depth strata categories were designed to best capture differences in benthic communities, to ensure consistent sampling across ecoregions and to maintain sufficient sample sizes within each depth strata [51]. At each site, a single depth stratum was surveyed (either shallow, mid or deep) to maximize spatial coverage around each island while also quantifying depth effects for each island. As such, ‘site’ and ‘depth’ were synonymous in this sampling design, with multiple depth strata replicates surveyed within each island. The number of unique site/depth combinations (hereafter referred to as ‘sampling station’) at each island was proportional to the amount of hardbottom area and the variance of coral density in previous years, such that more sites were sampled in strata with higher variance and more hardbottom (see the electronic supplementary material ST1 for the number of depth stratum replicates for each island). This approach was preferable to surveying all three depth strata at fewer sites at each island, as this would result in more limited spatial coverage around each island and therefore give a less accurate representation of island-mean condition. This sampling strategy maximizes the power to test for inter- rather than intra-island differences, which is the core goal of NOAA’s NCRMP sampling design. Although NOAA’s defined depth strata ranges may appear broad, most data in this study fell within a 6.1 m range or less within each depth stratum, with 70% of the data within each depth stratum falling within a 3−4 m depth range (see the electronic supplementary material, S1.2).

### (b) Quantifying benthic community composition across depths

At each sampling station around each island, the mean percentage cover of benthic groups was quantified by taking 30 digital images of the benthos (0.7–1 m^2^ per image) at 1 m increments along a 30 m transect. Coral Point Count with Excel extensions [52] was then used to overlay 10 random points onto each image (300 points per transect) and the benthic group under each point assigned to one of the following categories: scleractinian (hard) coral, CCA; turf algae, including highly cropped to thick turf mats; fleshy upright macroalgae, *Halimeda* spp. (a common upright calcifying macroalga in the Pacific); fleshy and calcified encrusting macroalgae and Alcyonacea (soft) coral. We used these broad benthic groups for the following reasons: (i) they capture known key functions on reefs (e.g. reef-building versus non-accreting) that respond in predictable ways to local human impacts [38]; (ii) they allowed for comparisons of benthic communities across our broad geographical sampling extent that would not be so comparable at finer taxonomic resolutions owing to regional differences in benthic diversity; and (iii) to maintain geographical comparability and taxonomic continuity with a number of foundational depth zonation studies on coral reefs, therefore allowing us to better re-visit the depth zonation paradigm on contemporary coral reefs.

### (c) Data analyses

To test for an effect of depth (categorical fixed factor with three levels; shallow: 0−6 m, mid: >6−18 m, and deep: >18−30 m), human population status of islands (binary fixed factor: ‘unpopulated’ and ‘populated’) and ecoregion (fixed factor with six levels: ecoregions listed above) on benthic community structure, we used a three-way permutational analysis of variance (PERMANOVA) [53]. To account for possible variations in disturbance histories, benthic community succession and biophysical conditions that might vary on an island-by-island basis independently of human population status and ecoregion, we included the random factor ‘island’ nested within both ecoregion and population status (see the electronic supplementary material ST4 for further details and justification of the model design). Benthic cover data were square root transformed to downweigh the influence of highly dominant benthic variables [54] (see the electronic supplementary material S1.4 for a sensitivity analysis comparing alternative data transformations). PERMANOVAs were performed on Euclidean distance matrices, using a permutation of residuals under a reduced model (*n* = 9999 permutations; for details on this method see Anderson & Ter Braak [55]). To account for the unbalanced sample design and the potential overlap among the terms regarding the individual portions of variation that they explain, we ran the PERMANOVA using Type I sums-of-squares, whereby each term was fitted sequentially after accounting for any previous terms in the model [56]. In our study design, we tested for the effect of depth on benthic community patterns, acknowledging that geography can impact these patterns and that human impacts can disrupt them too. Hence, the order of the terms listed in the model were: depth, ecoregion, population status. To explore the amount of overlap in the explained variability among terms, we re-ran the analysis changing the order of the terms to observe any differences in the results (see the electronic supplementary material S1.5 for a sensitivity analysis comparing the alternative order of terms).

PERMANOVA can be sensitive to differences in dispersion when comparing groups that have unequal sample sizes [57]. The test for homogeneity (obtained using PERMDISP; see Anderson [58]) was statistically significant when used to compare the within-group multivariate dispersions across all factors (*F* = 4.5024, *p* = 0.008). However, we examined a scatter plot of the mean distance-to-centroid per group versus sample size, and no trends or outliers were found; the range of mean distances-to-centroid was also comparable across different combinations of levels of the factors of interest (see the electronic supplementary material, S1.6). As such, we concluded that the inter-group variations in multivariate dispersion did not compromise the integrity of our results.

Where the global model was significant, we used planned contrasts between factor levels to test for differences in benthic community structure across depths and population status within each ecoregion (but not between ecoregions). To determine the benthic taxa most responsible for driving differences in community structure between our significant planned contrasts (i.e. where *p* ≤ 0.05), we used canonical analysis of principal coordinates (CAP) [59]. This constrained ordination technique aims to maximize variation in *a priori* determined groups (in our case ‘depth’ within ecoregions). Influential benthic taxa in each case were identified by calculating the correlation between the original benthic cover for each benthic group and the first two ordination axes and plotting these as vectors as a bi-plot on the CAP plot. To identify indicator groups, we selected those benthic variables where *r* ≥ 0.5 (i.e. those with the strongest correlations). All PERMANOVA and CAP analyses were computed using the PERMANOVA+add on [56] for Primer v7 [60].

## 3. Results

**Figure 2.**
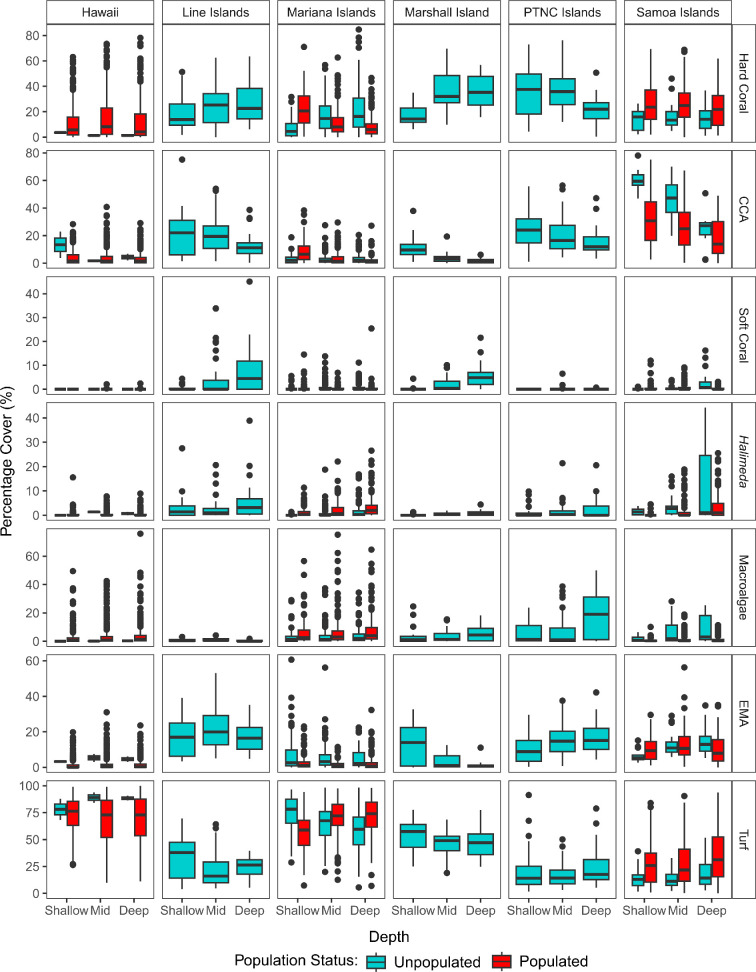
Variation in percentage cover of seven benthic groups: scleractinian (hard) coral, crustose coralline algae (CCA), soft coral, *Halimeda*, fleshy upright macroalgae (MA), encrusting macroalgae (EMA) and turf algae, between depth strata (shallow: 0−6 m, mid: >6−18 m, deep: >18−30 m) for unpopulated (blue) and populated (red) islands and atolls across six ecoregions in the Pacific Ocean. The central line within the box shows the median percentage cover, the upper and lower lines correspond to the first and third quartiles, respectively (25th and 75th percentiles). Whiskers show confidence intervals of 95% from the median, and dots indicate datapoints which are beyond the confidence intervals. Note different scales for percentage cover on the *y*-axes. Number of unpopulated versus populated islands within each ecoregion (and their respective number of survey sites in parentheses) is as follows. Hawaii Islands: unpopulated *n* = 1, populated *n* = 7 (6:755); Line Islands: unpopulated *n* = 3, populated *n* = 0 (98:0); Mariana Islands: unpopulated *n* = 8, populated *n* = 5 (354:473); Marshall Island: unpopulated *n* = 1, populated *n* = 0 (65:0); Phoenix, Tokelau and Northern Cook (PTNC) Islands: unpopulated *n* = 3, populated *n* = 0 (121:0); Samoa Islands: unpopulated *n* = 2, populated *n* = 3 (58:309). Total number of sampling stations (unique site/depth combinations) for each of the 33 islands and atolls (A) in our study are as follows (with number of depth strata replicates for shallow:mid:deep shown in parentheses): Niihau-Lehua 59 (20:21:18), Hawaii 124 (29:59:36), Kauai 74 (18:31:25), Lanai 87 (39:33:15), Maui 122 (37:57:28), Molokai 119 (36:44:39), Johnston (A) 6 (2:2:2), Alamagan 22 (4:10:8), Guguan 26 (6:10:10), Agrihan 20 (8:4:8), Sarigan 25 (6:10:9), Asuncion 53 (10:27:16), Farallon de Pajaros 30 (8:14:8), Maug 89 (14:50:25), Pagan 89 (18:33:38), Aguijan 29 (8:11:10), Guam 240 (84:81:75), Rota 61 (18:24:19), Saipan 98 (20:32:46), Tinian 45 (10:18:17), Wake (A) 65 (19:27:19), Baker 23 (5:11:7), Howland 36 (6:19:11), Swains 62 (20:22:20), Jarvis 42 (10:24:8), Kingman (A) 15 (3:9:5), Palymra (A) 41 (8:16:17), Rose (A) 56 (15:24:17), South Bank 2 (0:0:2), Ofu & Olosega 60 (17:21:22), Tau 46 (9:20:17), Tutuila 203 (46:85:72).

There was a significant three-way interaction between depth, ecoregion and population status on benthic community structure (PERMANOVA: pseudo-*F*_4_ = 1.7545, *p* < 0.001; table 1). As such, while benthic communities did vary with depth across our Pacific Ocean study system, these ecological gradients were context-specific and dependent on human population status and geography (figure 2). Switching the order of terms in the model had no significant effect, and the significant three-way interaction between depth, ecoregion and population status still held (see the electronic supplementary material, S1.5).

**Table 1.** Three-way permutational analysis of variance (PERMANOVA) testing for an effect of depth (three levels; shallow: 0−6 m, mid: >6−18 m, deep: >18−30 m), population status (two levels: unpopulated versus populated) and ecoregion (six levels: Hawaii Islands, Mariana Islands, Marshall Island, Phoenix, Tokelau and Northern Cook Islands, Line Islands and Samoa Islands) on coral reef benthic community structure across the Pacific Ocean (*n* = 2239 sites). (d.f., degrees of freedom.)

factor	pseudo-*F*	d.f.	*p*‐value
depth	6.51	2	0.0001
ecoregion	16.96	5	0.0001
population status	1.41	1	0.22
ecoregion x population status	3.23	2	0.06
depth x ecoregion	3.23	10	0.0001
depth x population status	9.50	2	0.0001
island (ecoregion x population status)	12.87	24	0.0001
depth x ecoregion x population status	3.02	4	0.04
depth x island (ecoregion x population status)	1.75	46	0.0001

**Figure 3.**
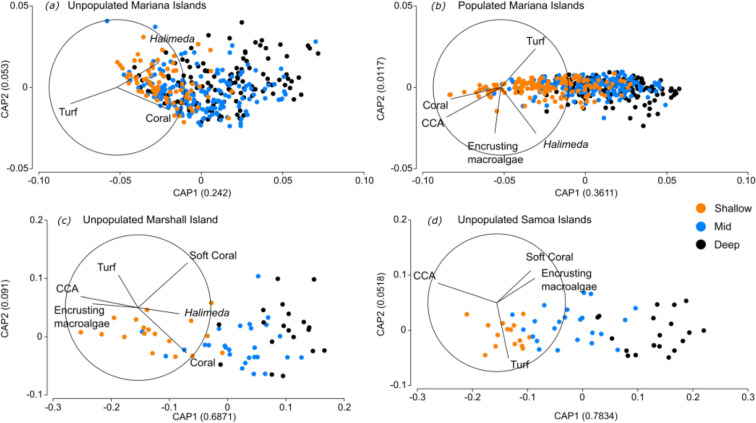
Canonical analysis of principal coordinates (CAP), showing changes in coral reef benthic community structure across depth strata (shallow: 0−6 m, mid: >6−18 m, deep: >18−3 0 m) for unpopulated (*a*) and populated (*b*) islands in the Mariana ecoregion and the unpopulated islands in the Marshall (*c*) and Samoa (*d*) ecoregions. These ecoregions were found to have significant differences in benthic community structure across depth strata (where *p* ≤ 0.05). The closer points appear, the more similar their benthic communities. Vector lines represent Pearson’s correlations (*r*) which are plotted for seven benthic groups: hard (scleractinian) coral, crustose coralline algae (CCA), soft coral, *Halimeda*, fleshy upright macroalgae, encrusting macroalgae and turf algae. The direction of the vector indicates the relationship of each benthic variable to the groupings in multivariate space, and the vector length is proportional to the strength of the correlation, with the circle representing *r* = 1. Benthic groups labelled are indicator groups, identified as the variables with the strongest correlations (*r* ≥ 0.5) with the first two CAP axes. See the electronic supplementary material S2.1 for full CAP results.

Planned contrasts across depths and population status within each ecoregion (but not between ecoregions) revealed a significant effect of depth on benthic community structure (*p* ≤ 0.05) at three of the six ecoregions (table 2). At both the unpopulated and populated islands of the Mariana ecoregion, differences in benthic community structure occurred between shallow and mid depths, and between shallow and deep depths. At the unpopulated islands in the Marshall and Samoa ecoregions, benthic communities differed across all depth strata (table 2). By contrast, there were no effects of depth observed at the populated and unpopulated islands of the Hawaii ecoregion and at the unpopulated islands of the Line and PTNC Islands ecoregions. For all ecoregions with a significant effect of depth, the greatest differences in benthic community structure occurred between shallow and deep depths and benthic communities differed the least between mid and deep depths (figure 3). There was a significant effect of population status on benthic community structure in the Samoa ecoregion. Here, differences in benthic communities occurred between all depth strata at unpopulated islands, but communities were not different across depth strata at populated islands (table 2). By contrast, the effects of population status were less consistent across the other five ecoregions.

**Table 2.** Permutational analysis of variance (PERMANOVA) planned contrasts, testing for the effects of depth (three levels; shallow (S): 0−6 m, mid (M): >6−18 m, deep (D): >18−30 m) and population status (two levels: unpopulated versus populated) on coral reef benthic community structure within each of six ecoregions (Hawaii Islands, Mariana Islands, Marshall Island, Phoenix, Tokelau and Northern Cook (PTNC) Islands, Line Islands and Samoa Islands) across the Pacific Ocean (*n* = 2239 sites). (Bold indicates significant difference between depth strata (*p* ≤ 0.05).)

ecoregion	unpopulated			populated	
	depth	*t*-statistic	*p*‐value	*t*-statistic	*p*‐value
Hawaii Islands	S-M	1.53	0.34	1.58	0.10
	S-D	1.02	0.67	1.18	0.26
	M-D	0.79	1.00	1.34	0.19
Mariana Islands	**S-M**	**2.98**	**0.001**	**2.99**	**0.01**
	**S-D**	**2.85**	**0.004**	**3.63**	**0.01**
	M-D	1.37	0.13	1.60	0.08
Marshall Island	**S-M**	**3.76**	**0.0001**		
	**S-D**	**4.23**	**0.0001**		
	**M-D**	**3.38**	**0.01**		
PTNC Islands	S-M	0.83	0.62		
	S-D	1.37	0.19		
	M-D	2.30	0.09		
Line Islands	S-M	1.24	0.13		
	S-D	1.16	0.34		
	M-D	1.25	0.24		
Samoa Islands	**S-M**	**2.13**	**0.003**	1.53	0.12
	**S-D**	**3.02**	**0.0001**	2.13	0.07
	**M-D**	**1.94**	**0.01**	1.87	0.09

**Figure 4.**
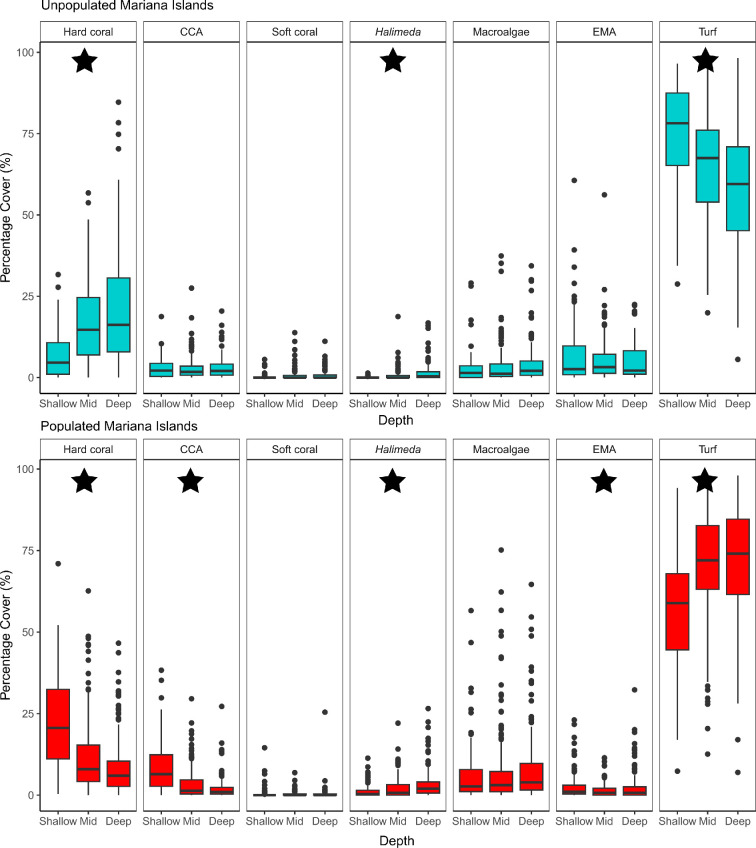
Percentage cover of seven benthic groups: hard (scleractinian) coral, crustose coralline algae (CCA), soft coral, *Halimeda*, fleshy upright macroalgae, encrusting macroalgae (EMA) and turf algae, across depth strata (shallow: 0−6 m, mid: >6−18 m, deep: >18−30 m) for the unpopulated and populated Mariana ecoregion. Box plots are shown for each depth stratum, showing median values (horizontal lines), boxes for values in the 25th−75th percentiles, vertical lines for values less than the 25th percentile and greater than the 75th and outliers (solid black circles). Black stars indicate benthic indicator groups (variables with the strongest correlations, *r* ≥ 0.5). At the unpopulated and populated islands of the Mariana ecoregion, significant differences (where *p* ≤ 0.05) were observed across shallow (0−6 m) to mid (>6−18 m) and shallow (0−6 m) to deep (>18−30 m) depth strata.

**Figure 5.**
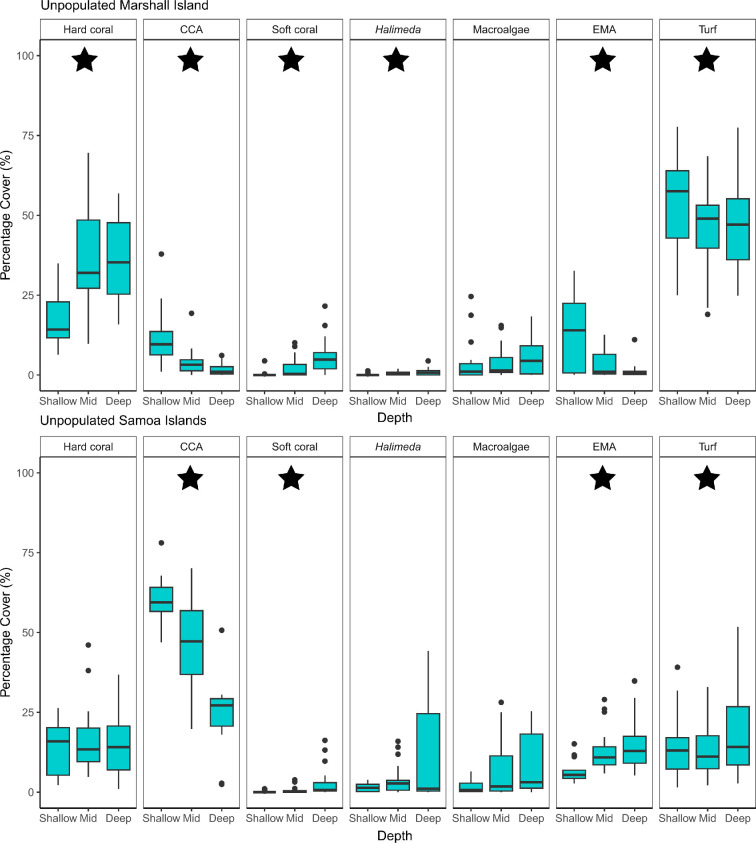
Percentage cover of seven benthic groups: hard (scleractinian) coral, crustose coralline algae (CCA), soft coral, *Halimeda*, fleshy upright macroalgae, encrusting macroalgae (EMA) and turf algae, across depth strata (shallow: 0−6 m, mid: >6−18 m, deep: >18−30 m) for the unpopulated Marshall and Samoa ecoregions. Box plots are shown for each depth stratum, showing median values (horizontal lines), boxes for values in the 25th−75th percentiles, vertical lines for values less than the 25th percentile and greater than the 75th and outliers (solid black circles). Black stars indicate benthic indicator groups (variables with the strongest correlations, *r* ≥ 0.5). At the unpopulated islands of the Marshall and Samoa ecoregions, significant differences in benthic community structure between all three depth strata were observed.

Where significant differences in benthic community structure existed across depths, the benthic groups driving these patterns of zonation varied across ecoregions and by population status. At the unpopulated Marshall Island, depth zonation was driven by a decreasing cover of CCA, encrusting macroalgae and turf algae cover with increasing depth, and an increasing cover of hard coral, soft coral and *Halimeda* with increasing depth (figures 3*c* and 5). Similarly, at the unpopulated islands of the Mariana ecoregion, depth zonation was driven by decreasing turf algae cover with increasing depth, and increasing hard coral and *Halimeda* cover with increasing depth (figures 3*a* and 4). Conversely, at the populated islands of the Mariana ecoregion, we observed the inverse trends in depth zonation—hard coral and CCA cover decreased with increasing depth, while turf algae and *Halimeda* cover increased with increasing depth. While encrusting macroalgae cover decreased with increasing depth, it then remained relatively consistent across the mid to deep depths (figures 3*b* and 4). Finally, at the unpopulated islands of the Samoa ecoregion, the key benthic groups driving depth zonation patterns were CCA, soft coral, encrusting macroalgae and turf algae. CCA cover decreased with increasing depth, while soft coral and encrusting macroalgae cover increased with increasing depth. Turf algae showed more complex patterns of depth zonation; deeper depths were characterized by higher turf cover, which then decreased in cover at mid depths, followed by an increase again at shallower depths (figures 3*d* and 5).

## 4. Discussion

Depth has long been acknowledged as a key determinant of coral reef benthic community structure [12–15]. However, the degree to which regional differences in biophysical processes govern and human activities might alter naturally occurring zonation patterns is unclear. Across our Pacific Ocean study system, depth zonation did not always occur at the taxonomic resolution of our data (broad benthic groups). In fact, we only saw evidence of depth zonation at three of the six ecoregions. Where depth zonation did exist, there was no universal ‘natural’ zonation pattern even in the absence of confounding local human impacts. The benthic groups most responsible for driving patterns of community depth zonation differed across geographies, and in some cases, the same benthic group showed different depth-related changes in percentage cover in one ecoregion versus another. We also found evidence of human-disrupted changes to benthic community depth zonation; patterns were inversed across depths and less distinct at populated compared to unpopulated islands within two of the ecoregions. Our findings add to a growing body of evidence that coral reef communities are naturally highly variable, but that human activities can also disrupt natural patterns of ecological organization.

Depth zonation on tropical coral reefs can be driven by gradients in biophysical conditions [16–18] and energetic resource supply [19–21]. We found an effect of depth on benthic community structure at the unpopulated and populated islands in the Mariana ecoregion and the unpopulated islands in the Marshall and Samoa ecoregions. At the populated islands in the Mariana ecoregion and the unpopulated islands in the Marshall and Samoa ecoregions, the cover of CCA tracked expected depth zonation patterns; peaking in the shallows and decreasing in cover with increasing depth. This is probably owing to CCA’s ability to photoacclimate and its wave-tolerant low-lying form, enabling it to occupy the well-lit but wave-exposed shallows [13,61]. A higher cover of CCA in shallow reef environments may also reflect herbivorous grazing pressure across the depth gradient [25,62], whereby rapidly colonizing CCA can outcompete turf when herbivore biomass is high [63–65]. In the ecoregions where we found an effect of depth on benthic community structure, the cover of soft coral and the calcified macroalga *Halimeda* also tracked expectations across the depth gradient; both increased with increasing depth. These patterns are probably owing to increased proximity to upwelled sources of nutrients and enhanced planktonic production at depth providing heterotrophic soft corals with food [66] and *Halimeda* capitalizing on the reprieve from surface wave stress and increased nutrient concentrations at depth [67,68]. Coral reef zonation studies often describe a peak in the cover of hard coral at intermediate depths (10-20 m), where there is a balance between food availability (light and plankton) and levels of physical disturbance [13,15,69]. This zonation pattern was not observed in our study. Instead, the cover of hard coral showed inconsistent patterns across depth strata between ecoregions and population status. We found that with increasing depth, hard coral cover increased, decreased or remained similar across depths depending on the ecoregion and population status. These variable patterns are perhaps owing to the mixotrophic strategy employed by most reef-building corals that allows them to occupy space throughout the depth gradient [21,28,70,71]. Hard corals also have flexible morphologies and predictable changes in coral assemblages with depth, with more wave-tolerant encrusting corals occupying the shallows and more structurally complex corals occupying deeper depths [17,24,29,30]. As such, patterns of overall hard coral cover can appear similar across depths or show inverse patterns in one location compared to another. Given that corals and other benthic groups in our study often have species-specific responses to gradients in biophysical conditions and energetic resource supply, future work should include increased taxonomic resolution of our broad benthic groups. A higher taxonomic resolution may reveal specific depth preferences for different species (or groups of species), aiding in the identification of zonation patterns and enhancing our understanding of depth zonation across our study system.

At the three ecoregions where we found evidence of depth zonation, there was no generalizable ‘natural’ zonation pattern. The benthic groups most responsible for driving patterns of community depth zonation differed across geographies, and in some cases, the same benthic group showed different depth-related changes in percentage cover in one ecoregion versus another. The Pacific islands in this study span large biophysical gradients known to influence benthic community structure, including surface wave energy, seawater temperature and primary production [45,46]. These gradients in biophysical drivers can create differences in the relative dominance of benthic groups and set natural bounds on their community organization [43]. It is reasonable to expect that these effects manifest across depths too and that the observed spatial variation in depth zonation patterns is, in part, driven by ecoregion-specific biophysical processes. For example, at the ecoregions where we found evidence of depth zonation, the unpopulated and populated islands of the Mariana ecoregion had the highest turf algae cover across all depths. In the Pacific, sea surface temperature variability and surface wave stress are generally greatest at northern latitudes [30,72]. Turf algae as a dominant benthic group in the Mariana ecoregion possibly reflects their increased competitive abilities in regions where biophysical drivers limit the recruitment and survival of key calcifying competitors, such as hard coral and CCA [24,30,69,73,74]. Comparably, we found the unpopulated islands in the Samoa ecoregion had less turf algae and greater cover of reef calcifiers across all depths. These patterns are potentially driven by reef calcifiers benefiting from the relatively stable monthly sea surface temperatures and lower surface wave energy stress in these lower latitude islands [72]. Additionally, the high cover of reef calicifers across all depths in the Samoa ecoregion is strongly correlated with elevated aragonite saturation state that creates conditions conducive for calcification [48]. To this effect, it is reasonable to expect that depth zonation patterns between ecoregions would be more similar between those ecoregions in closer proximity to each other, and therefore experience more similar surrounding biophysical conditions, than those further apart. This is reflected in our findings. The unpopulated Mariana and Marshall Island ecoregions were the only ecoregions to exhibit consistent benthic depth zonation patterns across all six ecoregions in our study, specifically a decrease in turf algae cover and an increase in hard coral and *Halimeda* cover with increasing depth. The spatial proximity of these two ecoregions to each other and their similar latitudes (figure 1) means they experience similar surrounding climatological ranges in biophysical conditions [45] which is probably contributing to the similarity in benthic community depth zonation patterns.

Anthropogenic impacts can reconfigure natural patterns of depth zonation in tropical coral reef communities [25]. We found evidence of human-disrupted changes to benthic community depth zonation in the Mariana ecoregion, where patterns were inversed across depths at populated compared to unpopulated islands. In the Mariana ecoregion, depth zonation at the unpopulated islands was driven by decreasing turf algae cover and increasing hard coral cover with increasing depth. Conversely, at the populated islands, hard coral cover decreased with increasing depth, while turf algae increased with increasing depth. Unexpectedly, we found higher coral cover in the shallows of the populated islands compared to unpopulated. Typically, under the presumption that local human impacts degrade reef calcifiers [38], we would expect to find lower coral cover in the shallows of populated islands compared to unpopulated. However, in some cases, local human impacts can create novel reef states, characterized by high overall coral cover but reduced diversity and a dominance of stress-tolerant and weedy coral species [75,76]. Although these novel community assemblages may retain high overall hard coral cover, they often do not maintain key ecosystem services [77,78]. An alternative hypothesis is that coral communities at these shallow reefs of the populated islands in the Mariana ecoregion have not undergone assemblage shifts to novel states, but are simply comprised of coral species more tolerant to direct local human impacts. Their resilience could mean that rather than reconfiguring in community composition, their dominant coral species are inherently better adapted to withstand chronic human disturbance, allowing them to survive where other, less tolerant species might not [75]. Testing these alternative hypotheses would require an increased taxonomic resolution of our broad benthic groups and would be an interesting focus for future work.

Local and global human impacts can lead to coral reef communities becoming less spatially variable [79–81]. In support of this, we found evidence of depth zonation across all depths at unpopulated islands, but not at populated islands in the Samoa ecoregion. Across our study system, herbivorous fishes are integral in supporting reef calcifier dominance over competitive fleshy algal cover [65,82]. However, herbivorous fish biomass is negatively affected by local human impacts, particularly fishing pressure [39,44,83]. Populated islands in the Samoa ecoregion experience higher fishing pressure and consequently have lower herbivorous reef fish biomass compared to unpopulated islands [39]. We hypothesize that this reduction in herbivorous fish biomass on populated Samoa islands may be facilitating rapidly colonizing turf algae to outcompete other benthic groups across depths, resulting in an increasingly similar community structure across depths and the loss of the depth zonation signal.

## 5. Future work

Our results show that local human impacts interact with geography to drive benthic community depth zonation on contemporary coral reefs. However, past disturbance events such as mass coral bleaching and tropical cyclones may also disproportionately impact shallower reef communities compared to those offered some reprieve in deeper waters, as well as impacting some ecoregions, islands or sections of island coastline more than others. In some cases, these historical disturbances may have driven shifts in benthic community structure across our study system that could partly confound our signals of depth zonation across ecoregions and population status, despite our inclusion of the ‘island’ random effect term in our statistical model (electronic supplementary material, ST4). For instance, the unpopulated island of Swains in the PTNC Islands ecoregion was heavily impacted by Cyclone Percy in 2005 [84] prior to our surveys taking place in 2010−2014. Since reefs can take a decade or more to recover to their pre-disturbance state following acute disturbance [85], the latent effects of such disturbances could still be visible in the benthic community. Future work could use case study examples of known past disturbances, like the cyclone at Swains, to better understand these events' lasting effects on coral reef benthic community depth zonation patterns. Some of our study islands were also exposed to acute disturbance following our survey period. For example, the unpopulated island of Jarvis in the Line Islands ecoregion and the populated island of Hawaii in the Hawaii ecoregion suffered mass bleaching-induced coral mortality following a marine heatwave associated with the 2015 El Niño [35,86]. These case studies offer opportunity in the future to learn about how global-scale human impacts, like climate change-induced ocean warming, may be reconfiguring and disrupting benthic community depth zonation patterns alongside more local human impacts. It is likely that these acute climate disturbances will further exaggerate the differences between historic ecological zonation paradigms and the reality of ecological organization on contemporary coral reefs, and it is crucial we quantify this change. Finally, owing to the lack of spatially resolved data on local human impacts on most coral reefs globally and the fact that local human population density is a poor predictor of these impacts [35], we used the binary definition of ‘unpopulated’ versus ‘populated’ to group our study islands and test for the effect of ‘population status’ on benthic community depth zonation. Future work should unpack how specific human impacts (e.g. wastewater pollution) and gradients in local management regimes (e.g. herbivorous fisheries management) affect benthic community depth zonation patterns on coral reefs. To do this, areas where the spatial and temporal gradients in local human impacts have been well resolved and quantified, like the west coastline of Hawaii Island [35], could be used as local case studies. Benthic community depth zonation patterns could be compared across gradients in these impacts, determining if there are specific levels of impact (i.e. thresholds) that induce the loss or inversion of the benthic community depth zonation signal.

## 6. Conclusion

Early descriptions of depth zonation patterns in coral reef communities generated the expectation that such patterns were somewhat ‘universal’ across contexts [12–15]. Here, however, we provide novel evidence of spatially dependent effects of depth on benthic community structure across the Pacific Ocean. Notably, we found that depth zonation did not always occur at the taxonomic resolution of our data. In instances where depth zonation patterns existed, individual benthic groups did not change consistently with depth across ecoregions, probably owing to geographical differences in biophysical conditions known to structure these communities. Furthermore, our results reveal benthic community depth-dependent zonation may be disrupted by local human impacts, but the extent by which communities are affected is not consistent across ecoregions and varies across depths. Our findings indicate that depth zonation patterns across our study system are highly context-specific and spatially heterogeneous, probably driven by the complex interactions of co-occurring biophysical and anthropogenic drivers [42]. As such, generalized patterns and ecological theories of zonation based on energetic resource supply or natural disturbance gradients should be applied cautiously to contemporary reefs. Overall, our study shows the importance of revisiting historic ecological paradigms to evaluate their geographical generality and contemporary relevance in this era of rapid change.

## Data Availability

Data can be obtained from the Dryad Digital Repository [87]. Supplementary material is available online [88].
